# Model-informed repurposing of eliglustat for treatment and prophylaxis of Shiga toxin-producing *Escherichia coli* hemolytic-uremic syndrome (STEC-HUS) in children

**DOI:** 10.1007/s00467-025-06688-3

**Published:** 2025-02-03

**Authors:** David F. G. J. Wolthuis, Jolien J. M. Freriksen, Mendy ter Avest, Reena V. Kartha, Saskia N. de Wildt, Kioa Wijnsma, Nicole C. A. J. van de Kar, Rob ter Heine

**Affiliations:** 1 Department of Internal Medicine, Radboudumc, Geert Grooteplein Zuid 10, 6525GA Nijmegen, The Netherlands; 2 Department of Pharmacy, Radboudumc, Nijmegen, The Netherlands; 3 Center for Orphan Drug Research, Department of Experimental and Clinical Pharmacology, University of Minnesota, Minneapolis, USA; 4 Department of Pediatric Nephrology, Amalia Children’s Hospital, Radboudumc, Nijmegen, The Netherlands; 5 Department of Intensive Care, Radboudumc, Nijmegen, The Netherlands; 6 Department of Neonatal and Pediatric Intensive Care, Erasmus MC, Rotterdam, The Netherlands

**Keywords:** Eliglustat, Repurposing, STEC-HUS, Physiologically based pharmacokinetic modeling, Population-based pharmacokinetic modeling

## Abstract

**Background:**

Shiga toxin-producing *Escherichia coli* hemolytic-uremic syndrome (STEC-HUS) is a severe illness predominantly affecting young children, with limited treatment options beyond supportive care. Eliglustat, approved for Gaucher disease, shows potential in reducing Shiga toxin binding to target glomerular endothelial cells in vitro, prompting interest as a treatment for STEC-HUS. However, it remains unknown what dose is likely to be effective and safe for treatment of STEC-HUS in the pediatric population. We hypothesize that effective and safe levels of eliglustat can be reached in children.

**Methods:**

We identified pharmacokinetic targets of efficacy for treatment and prophylaxis of STEC-HUS based on a preclinical model and human cardiac safety data. Then, we developed oral and intravenous dosing regimens using population pharmacokinetic (popPK) simulations based on an existing model enriched to allow extrapolation to a simulated virtual pediatric population. These dosing regimens were then confirmed using a verified physiologically based pharmacokinetic (PBPK) model.

**Results:**

We simulated, using popPK data, oral and intravenous dosing regimens resulting in adequate target exposure in > 90% of all patients, with minimal expected risk for cardiotoxicity. Confirmation of these dosing regimens with PBPK modeling resulted in very similar exposure, with lower interindividual variability and minimal toxicity potential.

**Conclusions:**

Based on pharmacokinetic modeling, we developed oral and intravenous eliglustat dosing regimens that are likely safe and effective for treatment of STEC-HUS and prophylaxis in case of outbreaks of STEC infections. Clinical evaluation of these dosing regimens in children suspected of or diagnosed with STEC-HUS is required and should include assessment of pharmacokinetics, efficacy, and safety (e.g., ECG monitoring).

**Graphical abstract:**

**Figure:**
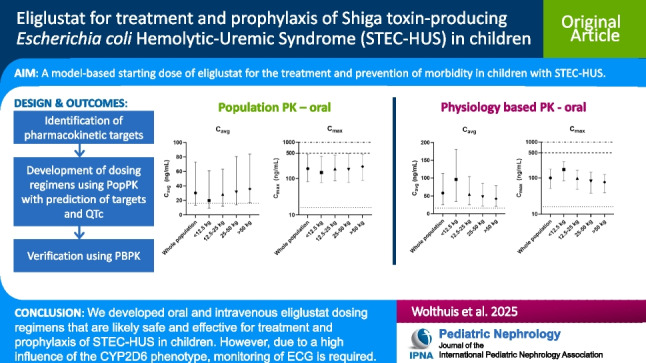
A higher resolution version of the Graphical abstract is available as Supplementary information

**Supplementary information:**

The online version contains supplementary material available at 10.1007/s00467-025-06688-3.

## Introduction

Shiga toxin-producing *Escherichia coli*–induced hemolytic-uremic syndrome (STEC-HUS) belongs to the most prevalent causes of acute kidney failure in pediatric patients [1]. STEC-HUS is classified within the group of thrombotic microangiopathies (TMA); hence, patients show the classical triad of mechanical hemolytic anemia, thrombocytopenia, and acute kidney failure. Dialysis is required in 50% of children during the acute phase and central nervous involvement, which occurs in 20 to 50% of children, and is the most serious complication which is associated with increased morbidity and mortality [1] with a mortality rate of 5% [2]. Supportive is the only treatment available [1]. The culprit in the pathophysiology of STEC-HUS is binding of Shiga toxin to globotriaosylceramide (G3b) receptor on (glomerular) endothelium after which retrograde transport of Shiga toxin causes inhibition of protein synthesis leading to apoptosis and ultimately the cascade leading to TMA [2, 3].

Interestingly, in patients with Gaucher disease, a liposomal storage disease, eliglustat is administered to inhibit biosynthesis of b-glucosylceramide, thereby also reducing the formation of Gb3 [4]. Eliglustat is a drug from the class of glucosylceramide synthase (GCS) inhibitors. Various studies showed a promising effect of eliglustat in the treatment of STEC-HUS in vitro [5, 6] and studies for the treatment of Gaucher with eliglustat are ongoing in children [7]. Due to the potential severe consequences of STEC-HUS, it is important to carefully determine the optimal dose of eliglustat to avoid subtherapeutic effects while preventing toxicity. Furthermore, administration of oral medication may be complicated in critically ill children. Therefore, a parenteral formulation will facilitate drug dosing. Our aim was, therefore, to develop a proposal for a safe and effective eliglustat dosing regimen for prophylaxis and treatment of STEC-HUS in the pediatric population.

Given the potential severity of STEC-HUS and the promising effects of eliglustat in vitro, determining the optimal dosing regimen is crucial for safety and efficacy, especially in pediatric patients. Pharmacokinetic (PK) modeling can help optimize drug dosing by simulating how the drug behaves in the body based on factors like age and genetics. Two common approaches in PK modeling are population pharmacokinetics (popPK) and physiologically based pharmacokinetic modeling (PBPK). PopPK examines variability in drug concentrations across populations, helping to tailor dosing for different patient groups, while PBPK uses physiological data to predict drug behavior in the body.

An important advancement is the use of virtual populations, which simulate diverse patient groups based on genetic, age, and disease-related variability. This approach allows for more accurate predictions of drug behavior, particularly in underrepresented populations like children or those with complex conditions. In this study, we use PK modeling with a virtual pediatric population to develop a safe and effective eliglustat dosing regimen for STEC-HUS, ensuring optimal dosing for both efficacy and safety.

## Methods

### General approach

**Fig. 1:**
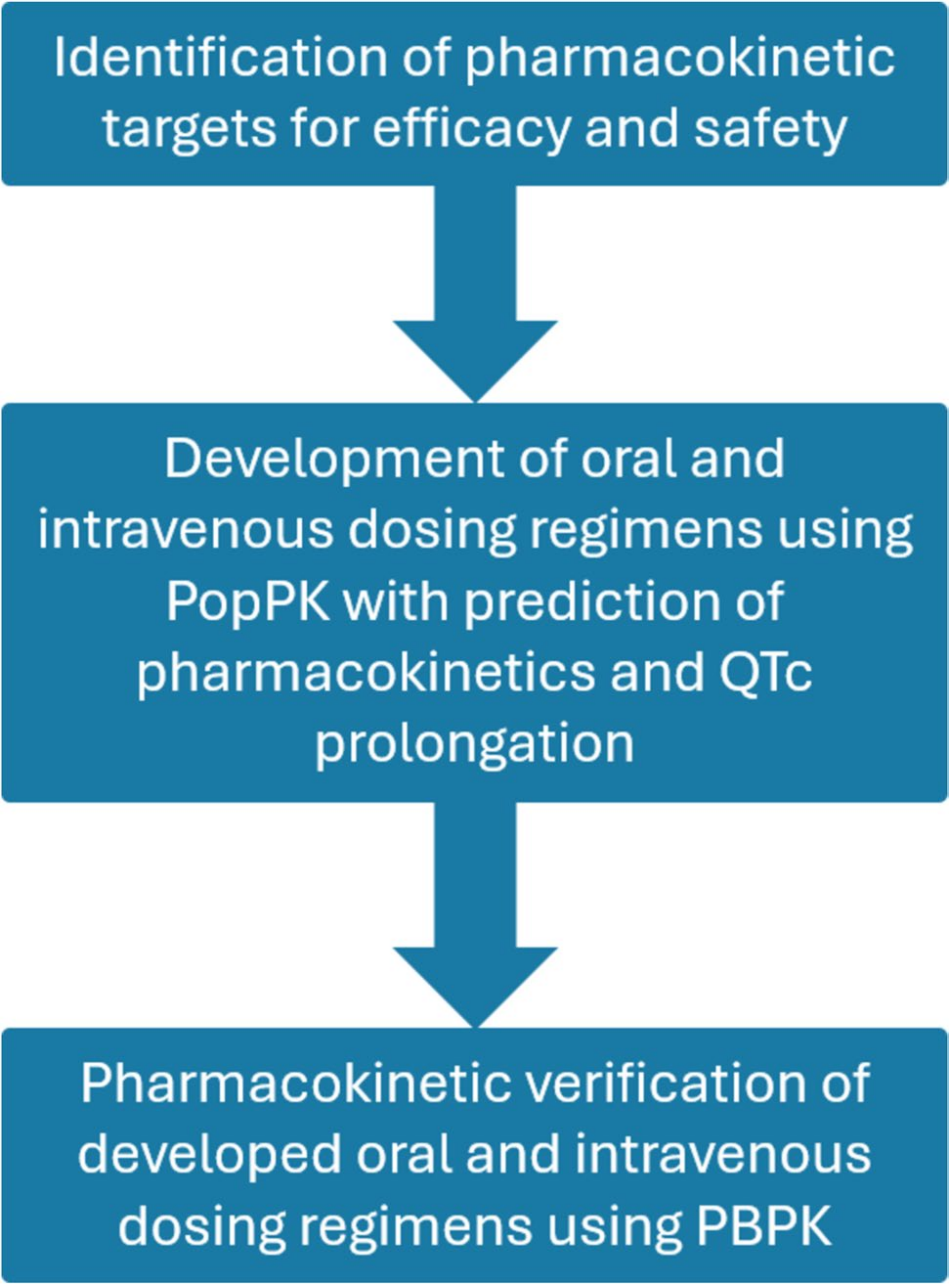
Schematic overview of the stepwise approach

Our general approach is depicted in Fig. 1. We first identified pharmacokinetic (PK) targets for efficacy and safety, based on the well-established relationships between exposure of eliglustat and in vitro efficacy for reducing endothelial Shiga toxin binding and in vivo toxicity (QTc prolongation). We then proposed a safe and effective dose of eliglustat for STEC-HUS in children. For this purpose, we first performed a population pharmacokinetic (PopPK) simulation to identify both an oral and an intravenous dosing regimen, which was then verified by means of physiologically based pharmacokinetic (PBPK) simulations.

### Rationale for pharmacokinetic targets

#### Pharmacokinetic efficacy target rationale

A plasma concentration of 16 ng/mL was selected as PK efficacy target for treatment and prophylaxis of STEC-HUS. At this concentration, eliglustat demonstrated the ability to diminish the binding of Shiga toxin to Gb3 at the cellular surface of various cell lines (human glomerular microvascular endothelial cells and human blood outgrowth endothelial cells) and it resulted in lower cellular levels of Gb3, as shown earlier in renal tubular epithelial cells [6, 8]. This PK efficacy target was chosen based on previous in vitro research indicating that an eliglustat plasma concentration of 8.0 nmol/L—which corresponds with 3.2 ng/mL, based on the molecular weight of eliglustat (404 g/mol)—led to a range of reduced Shiga toxin binding to Gb3, to reducing permanent damage of kidneys, brain, and intestinal tract [5]. Since 80% of eliglustat is bound to plasma proteins [9], a concentration of 16 ng/mL (corresponding to an unbound concentration of 3.2 ng/mL) was selected as PK efficacy target for this study.

#### Pharmacokinetic safety target rationale

Eliglustat, in supra-therapeutic doses, has a non-clinically relevant, albeit statistically significant, effect on cardiac repolarization that correlates with the plasma concentration of eliglustat [10]. The ENCORE study (eliglustat versus imiglucerase in adult patients with Gaucher disease) revealed that there was no clinically relevant prolongation of QTc (defined as QTc > 500 ms or increase in QTc > 60 ms) with plasma concentrations lower than 500 ng/mL [10]. Furthermore, during early clinical development of eliglustat in healthy adults, with high single doses (up to 30 mg/kg) or high multiple doses (up to 350 mg twice daily for 10 days), which were associated with plasma concentrations up to 1000 ng/mL, no QTc interval prolongation was evident [11]. These data are in line with data from an electrocardiographic study in adults, where single doses of 800 mg, with maximum concentrations up to 600 ng/mL, did not prolong QTc to a clinically significant degree [10]. Since clinically relevant QTc prolongation was not reached at very high systemic exposure, clear pharmacokinetic safety cut-offs remain unknown. We, therefore, used plasma concentrations of 500–1000 ng/mL as arbitrary conservative pharmacokinetic targets to start monitoring for QTc prolongation.

### Dosing regimen selection

Given the relatively short elimination half-life of eliglustat in adults [11], multiple oral daily doses (four times daily) were deemed essential to ensure maximum inhibition of Shiga toxin binding during a dosing interval, while limiting peak concentrations. The pediatric population was pragmatically divided in four weight groups of < 12.5 kg, 12.5–25 kg, 25–50 kg, and > 50 kg and doses were adjusted to reach similar exposure across these weight bands, based on the known allometric relationship between body size and pharmacokinetics, as well as logical divisions of the 84-mg capsule formulation. For the intravenous dosing regimen, a continuous infusion was investigated to reduce pharmacokinetic variability during dosing and prevent unwanted high peak concentrations. The selected dosing regimens for the intravenous route were 16.8 mg per 24 h (84 mg in 5 days) for patients with a body weight < 12.5 kg, 33.6 mg per 24 h (168 mg in 5 days) for patients with a body weight of 12.5–25 kg, 67.2 mg per 24 h (336 mg in 5 days) for patients with a body weight of 25–50 kg, and 100.8 mg per 24 h (504 mg in 5 days) for patients with a body weight > 50 kg. For all dosing regimens, a 5-day treatment course was selected considering the expected duration of Shiga toxin excretion by STEC [12].

Various weight band-based oral dosing regimens were evaluated to assure effective exposure, defined as average concentration (*C*_avg_) and maximum concentration (*C*_max_), above the PK efficacy target, while limiting plasma concentrations that might lead to QTc prolongation (> 500–1000 ng/mL). For the oral dosing regimens, the *C*_avg_ was the output to calculate the fractions of patients above efficacy target (> 16 ng/mL). The *C*_max_ was derived to calculate the fraction of patients above the efficacy target as well as the fraction of patients with a safe exposure (< 500–1000 ng/mL). For the intravenous dose, we only derived the *C*_avg_. Although the dosing regimen for eliglustat in adults with Gaucher disease depends on CYP2D6 phenotype (once daily in poor metabolizers versus twice daily for other phenotypes), this approach was not considered as genotyping is likely not feasible in the acute setting when immediate treatment is warranted. We deemed this a safe approach, since eliglustat has a very wide therapeutic window with doses tested safely in adults up to 20-fold higher than approved [11] and additionally, close clinical monitoring is available to discontinue treatment in case of acute toxicity in the setting of STEC-HUS.

### Population pharmacokinetic simulations

A PopPK model for eliglustat in adults, as previously developed by the market authorization holder of eliglustat, was used as a starting point [13, 14]. This PopPK model is a 2-compartmental disposition model describing the PK of eliglustat after intravenous administration and oral absorption. The effect of CYP2D6 phenotype and multiple dosing on eliglustat bioavailability as well as the effect of CYP2D6 phenotype on clearance were part of this model. This established model for adults was extrapolated to the pediatric population by allometric scaling of pharmacokinetics to total body weight [15, 16], and by accounting for enzyme maturation. Activity of CYP2D6 reaches full activity shortly after birth [17], but to fully account for all influencing factors, CYP2D6 maturation function was included using a previously established relationship as described with a Hill equation [18]. The PopPK simulations were performed using the software package NONMEM V7.4 (Icon, Dublin, Ireland). For the simulations, a dataset population of 1000 virtual Caucasian children aged 0–18 years was generated using PopGen [19] with the addition of a representative distribution of CYP2D6 phenotypes based on the Dutch population [20]. The simulated population consisted of 50% females and the median body size was 30.7 kg, with an interquartile range of 19.7 to 47.1 kg. The NONMEM code for the PopPK model is provided in the Supplemental Material.

### PBPK simulations

PBPK simulations were conducted using the Simcyp® Simulator (version 21; Certara, Sheffield, UK). Following a pragmatic PBPK modeling approach as described by Van der Heijden et al. [21], we used a previously validated PBPK model to verify our results from the PopPK/PD simulations [22]. Relevant default population models were available from the Simcyp software: the “Sim-healthy volunteer” model (reflecting an adult population) and the “Sim-paediatric” model (reflecting a pediatric population) [23].

#### PBPK model verification

The PBPK model used was previously externally validated for adults using oral pharmacokinetic data [22]. The model was not verified in children due to missing PK data. No external validation of prediction of intravenous pharmacokinetics has yet been performed. Therefore, we simulated eliglustat pharmacokinetics after a single 1-h intravenous infusion of 50 mg in 100 virtual adult individuals (10 trials with 10 subjects). This was compared to the previously reported *C*_avg_ and *C*_max_ for the same dose as reported by the market authorization holder [24].

#### PBPK modeling of untested dosing scenarios

A set of virtual trials with pediatric subjects was conducted, with dosing regimens as suggested by PopPK simulations. Table 1 shows characteristics of the simulations. In this study, 15 trials with 15 pediatric subjects were simulated to capture interindividual biological pharmacokinetic variability. The default CYP2D6 phenotype of the distribution is applied which corresponds roughly to the phenotype distribution as reported for the Dutch population [20].

**Table 1:** Characteristics of PBPK model simulations

Simulation no	Weight of the population	Proportion of females	Dosing strategy
#1	< 12.5 kg	0.5	Oral: 21 mg, 4 times daily
#2	12.5–25 kg	0.5	Oral: 42 mg, 4 times daily
#3	25–50 kg	0.5	Oral: 84 mg, 4 times daily
#4	> 50 kg	0.5	Oral: 126 mg, 4 times daily

## Results

### Population pharmacokinetic simulations

#### Oral dose development

**Fig. 2:**
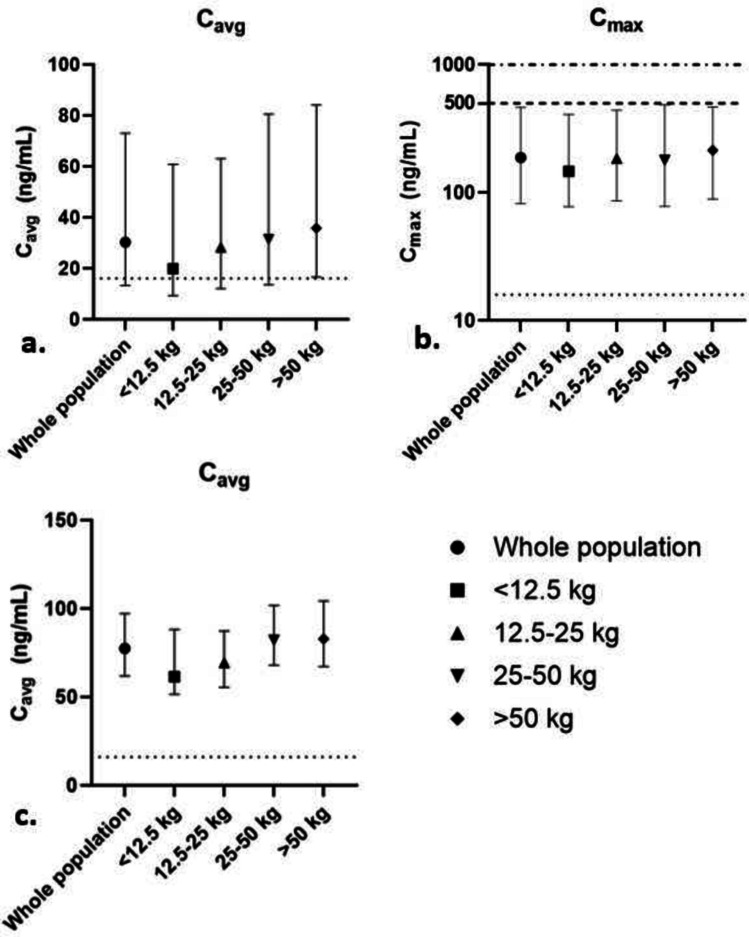
Predicted *C*_avg_ and *C*_max_ by PopPK modeling for distinct pediatric weight groups. **a** Predicted *C*_avg_ of the PopPK-informed oral dosing regimen. **b** Predicted *C*_max_ of the chosen oral dosing regimen^*^ using PopPK. **c** Predicted *C*_avg_ of the chosen continuous intravenous dosing regimen^#^ using PopPK.  indicates the predefined efficacy target (16 ng/mL),  indicates the predefined safety target of 500 ng/mL, and  indicates the safety target of 1000 ng/mL. The solid markers represent the median. The whiskers represent the predicted interquartile ranges. ^*^Proposed oral dosing regimen: 21 mg 4 times daily for patients < 12.5 kg, 42 mg 4 times daily for patients 12.5–25 kg, 84 mg 4 times daily for patients 25–50 kg, and 126 mg 4 times daily for patients > 50 kg. ^#^Proposed intravenous dosing regimen: 16.8 mg per 24 h (84 mg in 5 days) for patients < 12.5 kg, 33.6 mg per 24 h (168 mg in 5 days) for patients 12.5–25 kg, 67.2 mg per 24 h (336 mg in 5 days) for patients 25–50 kg, and 100.8 mg per 24 h (504 mg in 5 days) for patients > 50 kg. *C*_avg_, average plasma concentration; *C*_max_, maximum plasma concentration. Note the different *y*-axis scales

The selected dosing regimens per weight band were as follows: < 12.5 kg 21 mg 4 times per day, 12.5–25 kg 42 mg 4 times per day, 25–50 kg 84 mg 4 times per day, and > 50 kg 126 mg 4 times per day. Using these regimens, 69.1% had a *C*_avg_ and 97.4% a *C*_max_ above the threshold, indicating that pharmacologically relevant exposure can be reached in the majority of the population. The fraction of PK target attainment for efficacy was slightly lower in the youngest age group. These results are summarized in Table 2. In Fig. 2, median *C*_avg_ and *C*_max_ values during a 5-day treatment period are visualized. In total, 75.8% and 88.5% of the patients were predicted to reach a *C*_max_ below the respective arbitrary 500 and 1000 ng/mL targets which indicate that QTc monitoring might be considered.

**Table 2:** Summary of oral dose development by means of population pharmacokinetic simulations

	Fraction of patients with *C*_avg_ above 16 ng/mL	Fraction of patients with *C*_max_ above 16 ng/mL	Fraction of patients with *C*_max_ below 500 ng/mL	Fraction of patients with *C*_max_ below 1000 ng/mL
Whole population	69.1%	97.4%	75.8%	88.5%
< 12.5 kg 21 mg 4 times daily	58.1%	97.7%	76.7%	90.7%
12.5–25 kg 42 mg 4 times daily	66.6%	98.3%	76.0%	88.5%
25–50 kg 84 mg 4 times daily	70.0%	97.6%	75.4%	88.3%
> 50 kg 126 mg 4 times daily	75.6%	96.3%	76.0%	88.0%

Fraction of patients with *C*_avg_ above efficacy level, *C*_max_ above efficacy level, and *C*_max_ below safety level

*C*_*avg*_, average plasma concentration; *C*_*max*_, maximum plasma concentration

#### Intravenous dose development

**Fig. 3:**
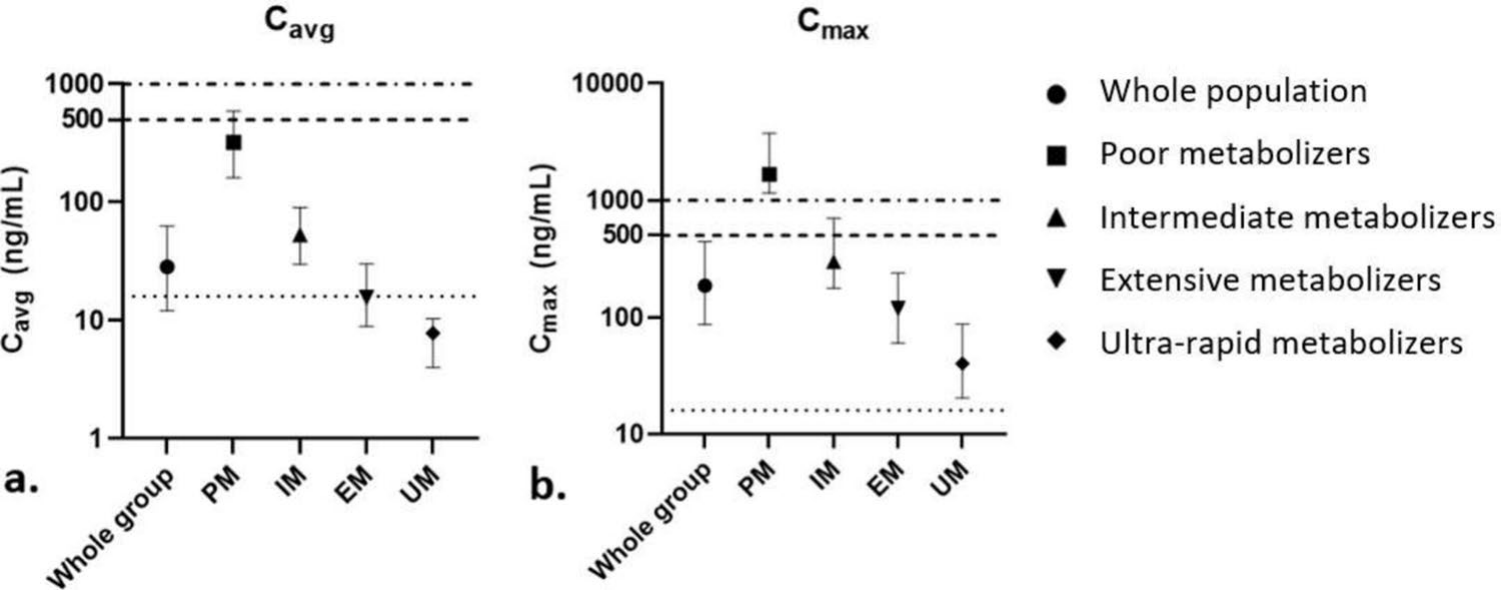
Predicted *C*_avg_ and *C*_max_ by PopPK modeling for distinct CYP2D6 phenotypes for weight group 12.5–25 kg. **a** Predicted *C*_avg_ of the chosen oral dosing regimen for the weight group 12.5–25 kg (42 mg 4 times daily) using PopPK. **b** Predicted *C*_max_ of the chosen oral dosing regimen for the weight group 12.5–25 kg (42 mg 4 times daily) using PopPK.  indicates the predefined efficacy target (16 ng/mL), and  and  indicate concentrations of 500 ng/mL and 1000 ng/mL as exposure to monitor for QTc prolongation. The solid markers represent the median. The whiskers represent the predicted interquartile ranges. *C*_avg_, average plasma concentration; *C*_max_, maximum plasma concentration; QTC_max_, maximum QT interval; PM, CYP2D6 poor metabolizer; IM, CYP2D6 intermediate metabolizer; EM, CYP2D6 extensive metabolizer; UM, CYP2D6 ultra-rapid metabolizer. Note the different *y*-axis scales

The predicted *C*_avg_ at day 5 are presented in Table 3. As observed, the fraction of patients above the proposed efficacy target was high at 100%. Furthermore, 100% of the population was predicted to stay below the safety target. Furthermore, we explored the predicted impact of CYP2D6 phenotype on PK. To illustrate this, we chose the weight band of 12.5–25 kg, because this is the weight band that most patients would presumably fall in, based on demographic data of STEC-HUS patients. As can be observed in Fig. 3, poor metabolizers (a very small fraction of the population) are predicted to have a relatively high exposure of eliglustat: 42.9% is predicted to have a *C*_avg_ above the predefined arbitrary safety targets of 500 ng/mL and 91.4% with a *C*_max_ above this threshold and for the threshold of 1000 ng/mL, this was 22.9% and 78.6%, respectively.

**Table 3:** Summary of intravenous dose development by means of population pharmacokinetic simulations

	Fraction of patients with *C*_avg_ above 16 ng/mL	Fraction of patients with *C*_avg_ below 500 ng/mL
Whole population	100%	100%
< 12.5 kg 84 mg in 5 days	100%	100%
12.5–25 kg 168 mg in 5 days	100%	100%
25–50 kg 336 mg in 5 days	100%	100%
> 50 kg 504 mg in 5 days	100%	100%

Fraction of patients with *C*_avg_ above efficacy and below safety level

*C*_*avg*_, average plasma concentration; *C*_*max*_, maximum plasma concentration

### PBPK model simulations

#### PBPK model verification for eliglustat pharmacokinetics after intravenous dosing in adults

The predicted adult intravenous clearance and *C*_max_ (mean ± standard deviation) of 85.4 ± 18.5 L/h and 101 ± 32 ng/mL respectively were well-aligned with reported human intravenous pharmacokinetic clearance of 85.8 ± 10.4 L/h and a *C*_max_ of 107 ± 25 ng/mL [24]. The most frequently applied criterion is the twofold acceptance range (i.e., the predicted-to-observed PK parameter ratios should be within 0.5 and 2 [25]), indicating excellent external validity of the PBPK model for prediction of eliglustat pharmacokinetics upon intravenous administration. Verification in children was not possible due to lack of data in this population.

#### Verification of developed PopPK dosing regimens with PBPK simulations

**Fig. 4:**
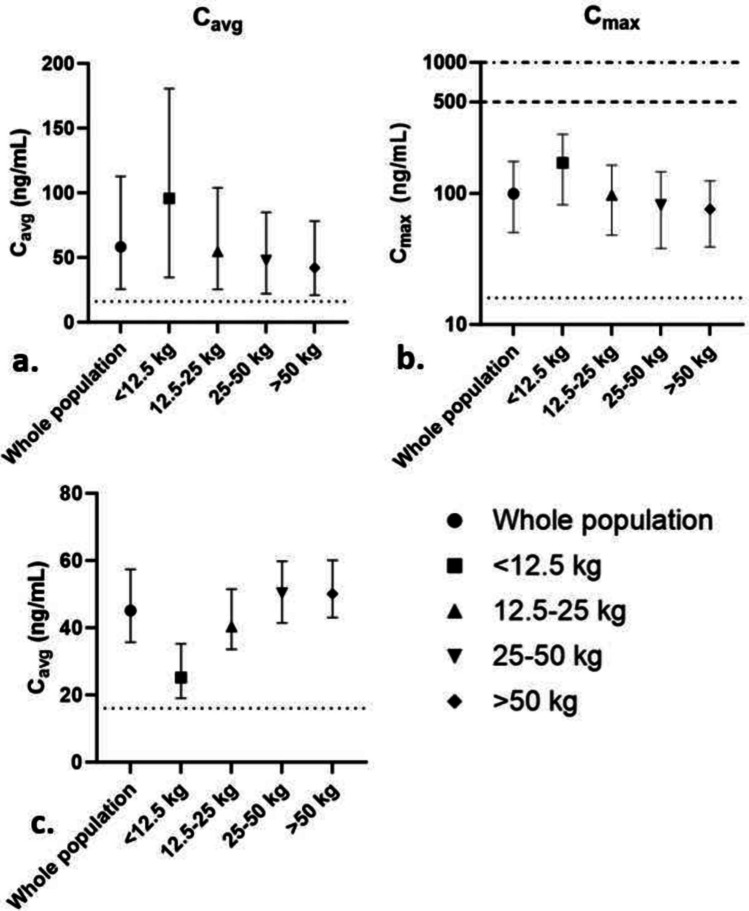
Predicted *C*_avg_ and *C*_max_ by PBPK modeling for distinct pediatric weight groups. **a** Predicted *C*_avg_ of the chosen oral dosing regimen^*^. **b** Predicted *C*_max_ of the chosen oral dosing regimen^*^. **c** Predicted *C*_avg_ of the chosen intravenous dosing regimen^#^.  indicates the predefined efficacy target (16 ng/mL), and  and  indicate concentrations of 500 ng/mL and 1000 ng/mL as exposure to monitor for QTc prolongation. The solid markers represent the median. The whiskers represent the predicted interquartile ranges. ^*^Proposed oral dosing regimen was 21 mg 4 times daily for patients with a body weight < 12.5 kg, 42 mg 4 times daily for patients with a body weight 12.5–25 kg, 84 mg 4 times daily for patients 25–50 kg, and 126 mg 4 times daily for patients > 50 kg. ^#^Proposed intravenous dosing regimen was 16.8 mg per 24 h (84 mg in 5 days) for patients with a body weight < 12.5 kg, 33.6 mg per 24 h (168 mg in 5 days) for patients with a body weight of 12.5–25 kg, 67.2 mg per 24 h (336 mg in 5 days) for patients with a body weight of 25–50 kg, and 100.8 mg per 24 h (504 mg in 5 days) for patients with a body weight of > 50 kg. *C*_avg_, average plasma concentration; *C*_max_, maximum plasma concentration. Note the different *y*-axis scales

The evaluation of the PopPK informed dosing regimens with PBPK modeling simulation showed similar, yet slightly higher eliglustat exposure in terms of average concentration and lower interindividual pharmacokinetic variability, leading to a larger proportion of patients with a predicted *C*_avg_ and *C*_max_ above the 16 ng/mL efficacy target (Tables 4 and 5). The predicted *C*_max_ remained below the defined safety threshold for most of the patients (Tables 4 and 5). The median and interquartile ranges of *C*_avg_ and *C*_max_ are shown in Fig. 4.

**Table 4:** Summary of oral dose development by means of physiologically based pharmacokinetic simulations

	Fraction of patients with *C*_avg_ above 16 ng/mL	Fraction of patients with *C*_max_ above 16 ng/mL	Fraction of patients with *C*_max_ below 500 ng/mL	Fraction of patients with *C*_max_ below 1000 ng/mL
Whole population	88.0%	95.2%	96.2%	98.6%
< 12.5 kg 21 mg 4 times daily	93.0%	98.4%	86.6%	95.2%
12.5–25 kg 42 mg 4 times daily	87.4%	95.7%	100%	100%
25–50 kg 84 mg 4 times daily	85.1%	94.6%	100%	100%
> 50 kg 126 mg 4 times daily	85.1%	93.6%	100%	100%

Fraction of patients with *C*_avg_ above efficacy level (16 ng/mL), *C*_max_ above efficacy level (16 ng/mL), and *C*_max_ below safety level (500 ng/mL)

*C*_*avg*_, average plasma concentration; *C*_*max*_, maximum plasma concentration

**Table 5:** Summary of intravenous dose development by means of physiologically based pharmacokinetic simulations

	Fraction of patients with *C*_avg_ above 16 ng/mL	Fraction of patients with *C*_avg_ below 500 ng/mL
Whole population	99.3%	100%
< 12.5 kg 84 mg in 5 days	94.2%	100%
12.5–25 kg 168 mg in 5 days	100%	100%
25–50 kg 336 mg in 5 days	100%	100%
> 50 kg 504 mg in 5 days	100%	100%

Fraction of patients with *C*_avg_ above efficacy level and below safety level

*C*_*avg*_, average plasma concentration; *C*_*max*_, maximum plasma concentration

## Conclusion and discussion

By means of pharmacokinetic simulations for purpose of pediatric extrapolation, we devised a likely safe dosing regimen that surpasses exposure predicted to clinically influence Shiga toxin binding. Despite substantial inter-individual variability in pharmacokinetics due to genetic variability of CYP2D6, most of the pediatric population is anticipated to achieve effective and safe eliglustat exposure with the proposed dosing regimens. This conclusion is further supported by the verification of our findings by PBPK modeling. One could argue that there is still much uncertainty regarding the real-world validity of our model predictions. It is crucial to note that our analyses were established using an extrapolated PopPK model developed by the market authorization holder as well as a previously developed and validated PBPK model, which was further verified by us for predictions of intravenous pharmacokinetics. During clinical development of eliglustat, PBPK modeling was to develop dosing regimens for different CYP2D6 genotypes and drug-drug interactions. These simulations led to label changes, without the necessity of performing a clinical study [10]. One may argue about the basis for us to carry out the proposed dose forward to a pediatric clinical study.

The systemic exposure (*C*_max_ and *C*_avg_) in children with the model-informed oral dosing regimen is predicted to be higher than exposure in adults with the approved adult dose. This was expected as the dose used for the highest weight band—approaching adults—at 126 mg four times daily is higher than the approved adult dose for Gaucher disease at 84 mg twice daily. Nonetheless, we expect these doses to be relatively safe. In a safety, tolerability, and pharmacokinetic study of eliglustat in adult healthy volunteers, the average *C*_max_ in a 12-day multiple dose study in the highest dose group (350 mg twice daily) was approximately 200–300 ng/mL, associated with an average increase in Fridericia’s QT of only 5 ms [11]. These results are in line with those of a thorough QT study of single-dose eliglustat [10]. In this study in the highest eliglustat dose group (800 mg), which was associated with a geometric mean *C*_max_ of 237 ng/mL, the associated increase in Fridericia’s QT was 5.7 ms (90% confidence interval 4.3–7.1 ms). The median predicted *C*_max_ for our dosing regimen was lower at approximately 200 ng/mL. As expected, eliglustat plasma levels are predicted to elevate in poor CYP2D6 metabolizers, which is only a small proportion (0.4–5.4%) of the population [26]. Immediate genotyping at presentation might help adjust the dose promptly when necessary, but rapid testing is currently not routinely available. It is important to note that the *CYP2D6* gene exhibits significant ethnic variability in allele frequencies, which can impact drug metabolism and response. While our simulation utilized a Caucasian-based dataset with genotypic data from the Dutch population, this choice may not fully reflect the genetic diversity observed in other ethnic groups. However, CYP2D6 poor metabolizers occur relatively frequently in Caucasians compared to other ethnicities, which decreases the risk of high levels of eliglustat in these other populations [26].

Although treatment with the model-informed dose is considered safe, there are still uncertainties about our predictions. A limitation may be that the models used in our analysis are mainly based on adult data in non-critically ill patients and healthy volunteers. Although allometric scaling and PBPK modeling is considered an appropriate method to scale adult pharmacokinetics to children, prospective evaluation of our predictions is pivotal [27]. Critical illness in children may alter the pharmacokinetics of a drug due to changes in absorption, distribution, metabolism, and excretion, which may result in higher or lower exposure than predicted and will likely result in increased pharmacokinetic variability [28–30]. Since the models we used have not been developed for critically ill patients, we should consider this as an additional layer of uncertainty for our predictions. Furthermore, we cannot rule out other sources of bias in predictions, e.g., due to age-dependent changes in bioavailability of an oral formulation [31]. Influence of CYP3A4 was not considered in the PopPK predictions. Although CYP3A4 only plays a small role in the metabolism of eliglustat, it should be noted that maturation of this enzyme may take up to 1 year after birth. In children < 1 year of age we, therefore, might underpredict systemic exposure to eliglustat in our PopPK simulations, but CYP3A only plays a very minor role in eliglustat metabolism. Another limitation of our study is that all predictions were made in absence of inhibitors of the metabolism of eliglustat, e.g., CYP2D6 and CYP3A inhibitors. We should take into account that critically ill children with STEC-HUS may have electrolyte imbalances in the setting of underlying TMA involving the kidneys, which in turn may impact cardiac repolarization and, thus, cardiac safety of co-administered drugs [32].

Taking all these uncertainties into account, we argue that the following safety measures should be taken in a clinical study in the target population: children with a long QTc at baseline (e.g., > 440 ms) and patients using strong inhibitors of CYP2D6 and CYP3A should not be administered eliglustat. Furthermore, patients being administered eliglustat should undergo frequent ECG monitoring. In case of QTc prolongation (e.g., QTc > 500 ms) during treatment or any other treatment-limiting toxicity, eliglustat should be discontinued. Furthermore, serum electrolytes should be closely monitored and corrected. Lastly, an intrapatient dose escalation trial may be used in a first cohort to allow each patient to reach a potential effective dose, while maximizing safety.

It should be noted that in critically ill children, protein binding may be decreased [33] resulting in lower total exposure than predicted, while the unbound plasma concentration, which is responsible for the pharmacological effect, is likely to be unaffected [34]. In future studies, in critically ill children, monitoring both total and plasma protein unbound concentrations of eliglustat is recommended to ensure PK target attainment.

In addition to QTc prolongation, other dose-dependent side effects of eliglustat are nervous system disorders (primarily headaches) and gastrointestinal disturbances, which may become more pronounced at higher doses [14]. Therefore, these adverse effects should be carefully monitored and included as key endpoints in clinical studies.

Furthermore, our findings suggest that eliglustat’s primary therapeutic potential lies in the early stages of STEC-HUS, where it may prevent or mitigate endothelial damage by reducing Gb3 synthesis and thereby limiting Shiga toxin activity. However, we recognize that in cases where significant endothelial injury and thrombotic microangiopathy are already established, the capacity of eliglustat to reverse these processes may be limited. This distinction underscores the importance of early intervention and highlights the need for complementary strategies to address advanced stages of the disease.

The commercially available oral formulations of eliglustat are capsules containing 100 mg eliglustat tartrate, corresponding with 84 mg of the active moiety eliglustat [35]. The proposed oral doses of 21 and 42 mg are not available. Eliglustat tartrate is considered a Biopharmaceutics Classification System (BCS) class I compound [14], meaning that the drug is characterized by high solubility and permeability, and the immediate release capsule formulation may likely be suspended in an aqueous solution in a syringe to facilitate administration of a lower dose. This approach can be employed temporarily until a pediatric formulation becomes available. A parenteral formulation is also not currently available nor approved. In the clinical development stage of eliglustat, an intravenous formulation was used to investigate the absolute bioavailability in healthy adults [14], indicating that development of a parenteral formulation is feasible.

In our analysis, we have focused on the GCS inhibitor eliglustat, but other GCS inhibitors like miglustat and venglustat might also be therapeutic options. However, miglustat is almost completely eliminated from the body by glomerular filtration. Since STEC-HUS is characterized by acute kidney damage, this complicates dosing. Another approved GCS inhibitor is venglustat, which has shown a better penetration of the blood–brain barrier than eliglustat [36]. This may be an advantage, since neurological symptoms occur in approximately 11% of STEC-HUS patients, indicating the importance of a drug’s ability to penetrate the brain, especially since Shiga toxin affects the central nervous system through Gb3 receptors localized in neurons and endothelial cells [37, 38].

A logical first step is to investigate PK, safety, and efficacy with the oral dosing regimens in the target pediatric population. With the data to define a dosage strategy of eliglustat, a proposal of a proof-of-concept study in children with STEC-HUS, investigating the total and plasma protein unbound pharmacokinetics, safety (with close ECG monitoring), and preliminary efficacy of the model-informed oral eliglustat dosing regimen, is the next step in the targeted treatment of STEC-HUS and in outbreaks of STEC infections to prevent the development of STEC-HUS.

## Supplementary Information

Below is the link to the electronic supplementary material.

Graphical abstract PPTX 196 KB)

Supplementary file2 (DOCX 20 KB)

## Data Availability

The data underlying this study are available upon request from the corresponding author.

## References

[1] Freedman SB, van de Kar N, Tarr PI (2023) Shiga toxin-producing Escherichia coli and the hemolytic-uremic syndrome. N Engl J Med 389:1402–1414 37819955 10.1056/NEJMra2108739

[2] Boyer O, Niaudet P (2022) Hemolytic-uremic syndrome in children. Pediatr Clin North Am 69:1181–1197 36880929 10.1016/j.pcl.2022.07.006

[3] Joseph A, Cointe A, Mariani Kurkdjian P, Rafat C, Hertig A (2020) Shiga toxin-associated hemolytic uremic syndrome: a narrative review. Toxins (Basel) 12:67 31973203 10.3390/toxins12020067 PMC7076748

[4] Belmatoug N, Di Rocco M, Fraga C, Giraldo P, Hughes D, Lukina E, Maison-Blanche P, Merkel M, Niederau C, Plӧckinger U, Richter J, Stulnig TM, Vom Dahl S, Cox TM (2017) Management and monitoring recommendations for the use of eliglustat in adults with type 1 Gaucher disease in Europe. Eur J Intern Med 37:25–32 27522145 10.1016/j.ejim.2016.07.011

[5] Feitz WJC, Bouwmeester R, van der Velden T, Goorden S, Licht C, van den Heuvel L, van de Kar N (2021) The Shiga toxin receptor globotriaosylceramide as therapeutic target in Shiga toxin E. coli mediated HUS. Microorganisms 9:2157 34683478 10.3390/microorganisms9102157 PMC8537462

[6] Sánchez DS, Fischer Sigel LK, Balestracci A, Ibarra C, Amaral MM, Silberstein C (2022) Eliglustat prevents Shiga toxin 2 cytotoxic effects in human renal tubular epithelial cells. Pediatr Res 91:1121–1129 34155339 10.1038/s41390-021-01622-3

[7] Veeva Systems (2018) Safety and efficacy of eliglustat with or without imiglucerase in pediatric patients with Gaucher disease (GD) type 1 and type 3 (ELIKIDS). https://ctv.veeva.com/study/safety-and-efficacy-of-eliglustat-with-or-without-imiglucerase-in-pediatric-patients-with-gaucher-di. Accesed Mar 2024

[8] Garimano N, Amaral MM, Ibarra C (2019) Endocytosis, cytotoxicity, and translocation of Shiga toxin-2 are stimulated by infection of human intestinal (HCT-8) monolayers with an hypervirulent E. coli O157:H7 lacking stx2 gene. Front Cell Infect Microbiol 9:396 31824869 10.3389/fcimb.2019.00396 PMC6881261

[9] Bennett LL, Turcotte K (2015) Eliglustat tartrate for the treatment of adults with type 1 Gaucher disease. Drug Des Devel Ther 9:4639–4647 26345314 10.2147/DDDT.S77760 PMC4554398

[10] Ruskin JN, Ortemann-Renon C, Msihid J, Ross L, Puga AC, Peterschmitt MJ, Cox GF, Maison-Blanche P (2020) How a concentration-effect analysis of data from the eliglustat thorough electrocardiographic study was used to support dosing recommendations. Mol Genet Metab 131:211–218 33012655 10.1016/j.ymgme.2020.09.003

[11] Peterschmitt MJ, Burke A, Blankstein L, Smith SE, Puga AC, Kramer WG, Harris JA, Mathews D, Bonate PL (2011) Safety, tolerability, and pharmacokinetics of eliglustat tartrate (Genz-112638) after single doses, multiple doses, and food in healthy volunteers. J Clin Pharmacol 51:695–705 20864621 10.1177/0091270010372387

[12] Bruyand M, Mariani-Kurkdjian P, Gouali M, de Valk H, King LA, Le Hello S, Bonacorsi S, Loirat C (2018) Hemolytic uremic syndrome due to Shiga toxin-producing Escherichia coli infection. Med Mal Infect 48:167–174 29054297 10.1016/j.medmal.2017.09.012

[13] Lu Q, Gao Y, Li J, Kanamaluru V (2018) Population pharmacokinetics of eliglustat in patients with Gaucher disease type 1 (GD1). J Pharmacokinet Pharmacodyn 45(Suppl 1):W-085

[14] FDA Center For Drug Evaluation And Research (2014) Clinical pharmacology review(s). Eliglustat Tartrate (Cerdelga). https://www.accessdata.fda.gov/drugsatfda_docs/nda/2014/205494orig1s000clinpharmr.pdf. Accesed Jan 2024

[15] Samant TS, Mangal N, Lukacova V, Schmidt S (2015) Quantitative clinical pharmacology for size and age scaling in pediatric drug development: a systematic review. J Clin Pharmacol 55:1207–1217 26009792 10.1002/jcph.555

[16] Johnson TN, Ke AB (2021) Physiologically based pharmacokinetic modeling and allometric scaling in pediatric drug development: where do we draw the line? J Clin Pharmacol 61(Suppl 1):S83-s93 34185901 10.1002/jcph.1834

[17] Allegaert K, van Schaik RH, Vermeersch S, Verbesselt R, Cossey V, Vanhole C, van Fessem M, de Hoon J, van den Anker JN (2008) Postmenstrual age and CYP2D6 polymorphisms determine tramadol o-demethylation in critically ill neonates and infants. Pediatr Res 63:674–679 18317231 10.1203/PDR.0b013e31816ff712

[18] T’Jollyn H, Snoeys J, Vermeulen A, Michelet R, Cuyckens F, Mannens G, Van Peer A, Annaert P, Allegaert K, Van Bocxlaer J, Boussery K (2015) Physiologically based pharmacokinetic predictions of tramadol exposure throughout pediatric life: an analysis of the different clearance contributors with emphasis on CYP2D6 maturation. AAPS J 17:1376–1387 26209290 10.1208/s12248-015-9803-z PMC4627449

[19] McNally K, Cotton R, Hogg A, Loizou G (2014) PopGen: a virtual human population generator. Toxicology 315:70–85 23876857 10.1016/j.tox.2013.07.009

[20] Zhou Y, Ingelman-Sundberg M, Lauschke VM (2017) Worldwide distribution of cytochrome P450 alleles: a meta-analysis of population-scale sequencing projects. Clin Pharmacol Ther 102:688–700 28378927 10.1002/cpt.690 PMC5600063

[21] van der Heijden JEM, Freriksen JJM, de Hoop-Sommen MA, Greupink R, de Wildt SN (2023) Physiologically-based pharmacokinetic modeling for drug dosing in pediatric patients: a tutorial for a pragmatic approach in clinical care. Clin Pharmacol Ther 114:960–971 37553784 10.1002/cpt.3023

[22] Sahasrabudhe SA, Cheng S, Al-Kofahi M, Jarnes JR, Weinreb NJ, Kartha RV (2022) Physiologically-based pharmacokinetic model development, validation, and application for prediction of eliglustat drug-drug interactions. Clin Pharmacol Ther 112:1254–1263 36056771 10.1002/cpt.2738 PMC9828395

[23] Johnson TN, Rostami-Hodjegan A, Tucker GT (2006) Prediction of the clearance of eleven drugs and associated variability in neonates, infants and children. Clin Pharmacokinet 45:931–956 16928154 10.2165/00003088-200645090-00005

[24] Australian Government Department of Health (2015) Australian Public Assessment Report: eliglustat (as tartrate) - Attachment 2. https://www.tga.gov.au/sites/default/files/auspar-eliglustat-150818.pdf. Accesed Jan 2024

[25] Sager JE, Yu J, Ragueneau-Majlessi I, Isoherranen N (2015) Physiologically based pharmacokinetic (PBPK) modeling and simulation approaches: a systematic review of published models, applications, and model verification. Drug Metab Dispos 43:1823–1837 26296709 10.1124/dmd.115.065920 PMC4613950

[26] Gaedigk A, Sangkuhl K, Whirl-Carrillo M, Klein T, Leeder JS (2017) Prediction of CYP2D6 phenotype from genotype across world populations. Genet Med 19:69–76 27388693 10.1038/gim.2016.80 PMC5292679

[27] Mahmood I, Tegenge MA (2019) A comparative study between allometric scaling and physiologically based pharmacokinetic modeling for the prediction of drug clearance from neonates to adolescents. J Clin Pharmacol 59:189–197 30192373 10.1002/jcph.1310

[28] Vet NJ, Brussee JM, de Hoog M, Mooij MG, Verlaat CW, Jerchel IS, van Schaik RH, Koch BC, Tibboel D, Knibbe CA, de Wildt SN (2016) Inflammation and organ failure severely affect midazolam clearance in critically ill children. Am J Respir Crit Care Med 194:58–66 26796541 10.1164/rccm.201510-2114OC

[29] Hartman SJF, Brüggemann RJ, Orriëns L, Dia N, Schreuder MF, de Wildt SN (2020) Pharmacokinetics and target attainment of antibiotics in critically ill children: a systematic review of current literature. Clin Pharmacokinet 59:173–205 31432468 10.1007/s40262-019-00813-w PMC7007426

[30] Boucher BA, Wood GC, Swanson JM (2006) Pharmacokinetic changes in critical illness. Crit Care Clin 22:255–271 16677999 10.1016/j.ccc.2006.02.011

[31] Nicolas JM, Bouzom F, Hugues C, Ungell AL (2017) Oral drug absorption in pediatrics: the intestinal wall, its developmental changes and current tools for predictions. Biopharm Drug Dispos 38:209–230 27976409 10.1002/bdd.2052 PMC5516238

[32] Harkins V, McAllister D, Reynolds B (2020) Shiga-toxin E. coli hemolytic uremic syndrome: review of management and long-term outcome. Current Pediatr Rep 8:16–25

[33] Thakkar N, Salerno S, Hornik CP, Gonzalez D (2017) Clinical pharmacology studies in critically ill children. Pharm Res 34:7–24 27585904 10.1007/s11095-016-2033-y PMC5177463

[34] Benet LZ, Hoener B-A (2002) Changes in plasma protein binding have little clinical relevance. Clin Pharmacol Ther 71:115–121 11907485 10.1067/mcp.2002.121829

[35] Balwani M, Burrow TA, Charrow J, Goker-Alpan O, Kaplan P, Kishnani PS, Mistry P, Ruskin J, Weinreb N (2016) Recommendations for the use of eliglustat in the treatment of adults with Gaucher disease type 1 in the United States. Mol Genet Metab 117:95–103 26387627 10.1016/j.ymgme.2015.09.002

[36] Wilson MW, Shu L, Hinkovska-Galcheva V, Jin Y, Rajeswaran W, Abe A, Zhao T, Luo R, Wang L, Wen B, Liou B, Fannin V, Sun D, Sun Y, Shayman JA, Larsen SD (2020) Optimization of eliglustat-based glucosylceramide synthase inhibitors as substrate reduction therapy for Gaucher disease type 3. ACS Chem Neurosci 11:3464–3473 33035424 10.1021/acschemneuro.0c00558 PMC7919060

[37] Costigan C, Raftery T, Carroll AG, Wildes D, Reynolds C, Cunney R, Dolan N, Drew RJ, Lynch BJ, O’Rourke DJ, Stack M, Sweeney C, Shahwan A, Twomey E, Waldron M, Riordan M, Awan A, Gorman KM (2022) Neurological involvement in children with hemolytic uremic syndrome. Eur J Pediatr 181:501–512 34378062 10.1007/s00431-021-04200-1 PMC8821508

[38] Obata F, Tohyama K, Bonev AD, Kolling GL, Keepers TR, Gross LK, Nelson MT, Sato S, Obrig TG (2008) Shiga toxin 2 affects the central nervous system through receptor globotriaosylceramide localized to neurons. J Infect Dis 198:1398–1406 18754742 10.1086/591911 PMC2684825

