# Supplementary material for: Model-informed repurposing of eliglustat for treatment and prophylaxis of Shiga toxin-producing Escherichia coli hemolytic-uremic syndrome (STEC-HUS) in children
Source: Pediatr Nephrol. 2025 Feb 3;40(6):2009–19. doi: 10.1007/s00467-025-06688-3 (PMC12031897; doi:10.1007/s00467-025-06688-3)
Supplement: Supplementary file 1 — Graphical abstract PPTX 196 KB) [file 467_2025_6688_MOESM1_ESM.pptx]

## Slide 1
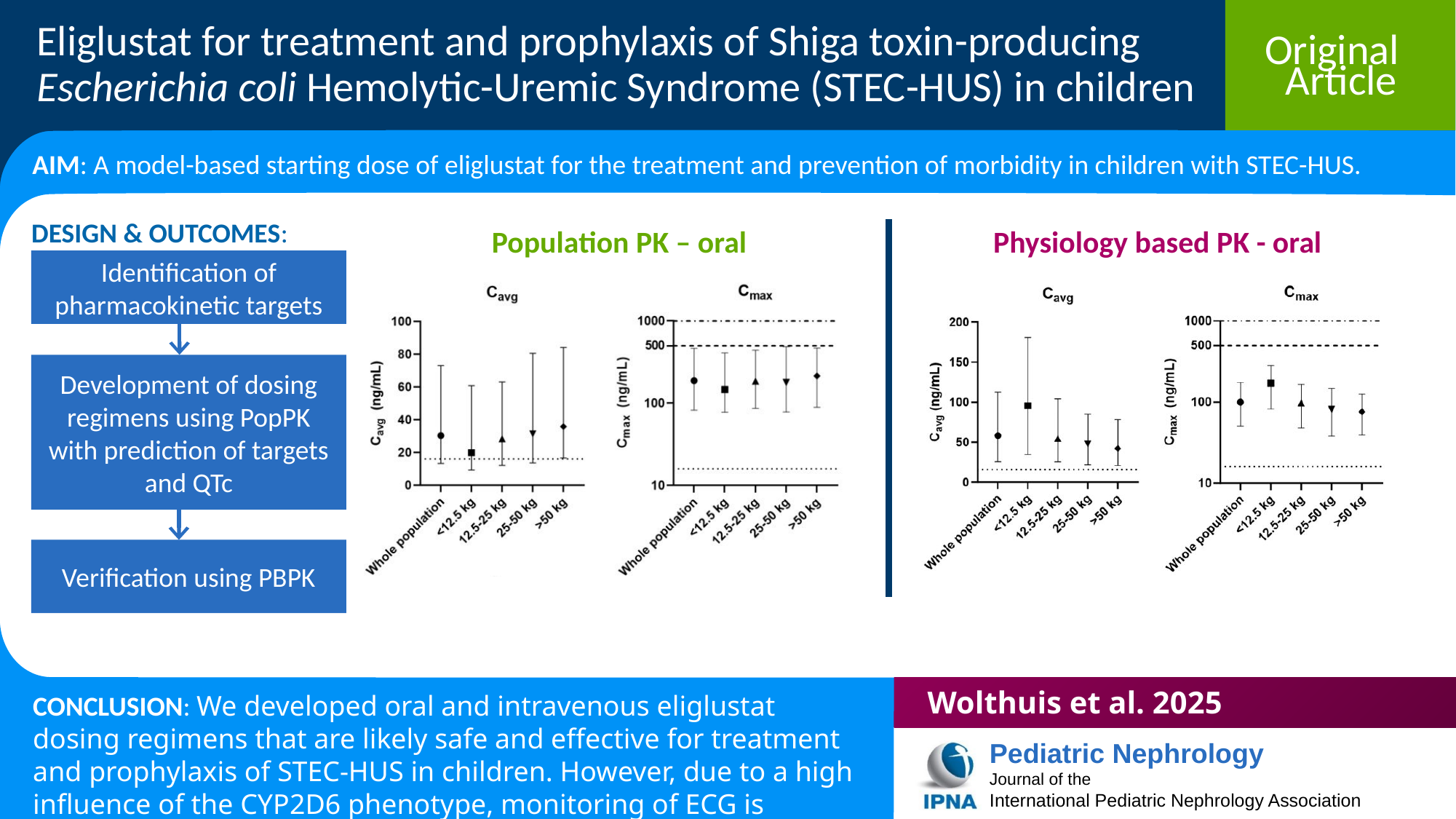

Eliglustat for treatment and prophylaxis of Shiga toxin-producing Escherichia coli Hemolytic-Uremic Syndrome (STEC-HUS) in children
AIM: A model-based starting dose of eliglustat for the treatment and prevention of morbidity in children with STEC-HUS.
DESIGN & OUTCOMES:
Physiology based PK - oral
Population PK – oral
Identification of pharmacokinetic targets
Development of dosing regimens using PopPK with prediction of targets and QTc
Verification using PBPK
Wolthuis et al. 2025
CONCLUSION: We developed oral and intravenous eliglustat dosing regimens that are likely safe and effective for treatment and prophylaxis of STEC-HUS in children. However, due to a high influence of the CYP2D6 phenotype, monitoring of ECG is required.
