# Supplementary material for: Model-informed repurposing of eliglustat for treatment and prophylaxis of Shiga toxin-producing Escherichia coli hemolytic-uremic syndrome (STEC-HUS) in children
Source: Pediatr Nephrol. 2025 Feb 3;40(6):2009–19. doi: 10.1007/s00467-025-06688-3 (PMC12031897; doi:10.1007/s00467-025-06688-3)
Supplement: Supplementary file 2 — Supplementary file2 (DOCX 20 KB) [file 467_2025_6688_MOESM2_ESM.docx]

**Supplemental material – NONMEM control stream**

$PROBLEM ELIGLUSTAT

$SUBROUTINE ADVAN6 TOL=3

$ABBREVIATED COMRES=3

$MODEL COMP=(DOSE) COMP=(CENTRAL) COMP=(PERIPHERAL) COMP=(AUC) COMP=(TABOVE)

$PK

IF(NEWIND.LE.1) THEN

COM(1) = -1

COM(2) = -1

COM(3) = -1

ENDIF

; --- TAD

IF (NEWIND.LE.1) THEN

DOSE=0

TDOS=0

ENDIF

;Remember dose and time of dose

IF (AMT.GT.0) THEN

DOSE=AMT

TDOS=TIME

ENDIF

;Time after dose for every record

TD2=TIME-TDOS

IF (TD2.GT.20) TAD=0

IF (TD2.LE.20) TAD=TD2

IF (OCC.EQ.1) IOV=ETA(1)

IF (OCC.EQ.2) IOV=ETA(2)

IF (OCC.EQ.3) IOV=ETA(3)

IF (OCC.EQ.4) IOV=ETA(4)

IF (OCC.EQ.5) IOV=ETA(5)

IF (OCC.EQ.6) IOV=ETA(6)

IF (OCC.EQ.7) IOV=ETA(7)

IF (OCC.EQ.8) IOV=ETA(8)

IF (OCC.EQ.9) IOV=ETA(9)

IF (OCC.EQ.10) IOV=ETA(10)

IF (OCC.EQ.11) IOV=ETA(11)

IF (OCC.EQ.12) IOV=ETA(12)

IF (OCC.EQ.13) IOV=ETA(13)

IF (OCC.EQ.14) IOV=ETA(14)

IF (OCC.EQ.15) IOV=ETA(15)

IF (OCC.EQ.16) IOV=ETA(16)

IF (OCC.EQ.17) IOV=ETA(17)

IF (OCC.EQ.18) IOV=ETA(18)

IF (OCC.EQ.19) IOV=ETA(19)

IF (OCC.EQ.20) IOV=ETA(20)

; -- BIOAVAILABILITY

IF (MIXNUM.EQ.1) PHENO=1 ; PM

IF (MIXNUM.EQ.2) PHENO=2 ; IM

IF (MIXNUM.EQ.3) PHENO=3 ; EM

IF (MIXNUM.EQ.4) PHENO=4 ; UM / UR

CHRONIC=0

IF (TIME.GT.168) CHRONIC=1 ; CHRONIC DOSING = >7 DAYS

IF (PHENO.EQ.3) FA=THETA(1)*(THETA(6)**CHRONIC) ; EM F1

IF (PHENO.EQ.1) FA=THETA(1)*THETA(2)*(THETA(5)**CHRONIC) ; PM F1

IF (PHENO.EQ.2) FA=THETA(1)*THETA(3)*(THETA(6)**CHRONIC) ; IM F1

IF (PHENO.EQ.4) FA=THETA(1)*THETA(4)*(THETA(6)**CHRONIC) ; UM F1

F1=FA*EXP(IOV)*EXP(ETA(21))

; --- ABSORPTION KA ALLOMETRICALLY SCALED

D1=THETA(7)*EXP(ETA(22))

KA=THETA(8)*((WT/70)**(-0.25))*EXP(ETA(23))

; --- CLEARANCE AND VOLUME ALLOMETRICALLY SCALED AND

; --- MATURATION OF CYP2D6 BASED ON https://link.springer.com/content/pdf/10.1208/s12248-015-9803-z.pdf

; --- AND MATURATION BASED ON https://static-content.springer.com/esm/art%3A10.1208%2Fs12248-015-9803-z/MediaObjects/12248_2015_9803_MOESM1_ESM.docx

CLBASE=THETA(9)

CLALLO=((WT/70)**0.75)

PMA=(AGE*52.18)+40 ; CALCULATION OF PMA IN WEEKS FRM AGE IN YEARS

CLMATUR=1/(1+((PMA/THETA(10))**THETA(11))) ; THETA(10) TM50 40.3 WEEKS, THETA(11) HILL 9.09 MATURATION FUNCTION FOR CYP2D6-MEDIATED METABOLISM

; -- EFFECT OF POOR METABOLIZER ON CL/VC

PM=0

IF (PHENO.EQ.1) PM=1

CLPM=THETA(12)**PM

; --- CLEARANCE

CL=CLBASE*CLALLO*CLMATUR*CLPM*EXP(ETA(24))

VC=THETA(13)*(WT/70)*EXP(ETA(25))

VP=THETA(14)*(WT/70)

Q=THETA(15)*CLALLO

S2=VC

K12=KA

K23=VC/Q

K32=VP/Q

K20=CL/VC

$DES

DADT(1) = -A(1)*KA

DADT(2) = A(1)*KA + A(3)*K32 -A(2)*(K20+K23)

DADT(3) = A(2)*K23-A(3)*K32

CONT1 = A(2)/S2

IF(CONT1.GT.COM(1)) THEN

COM(1)= CONT1

COM(2)= T

ENDIF

FLAGABOVE=0

IF (CONT1.GT.0.01616) FLAGABOVE=1

DADT(4)=CONT1

DADT(5)=FLAGABOVE

$MIX

; --- CYP2D6 PHENOTYPES FREQUENCY https://www.knmp.nl/downloads/g-standaard/farmacogenetica/achtergrondtekst-Farmacogenetica-CYP2D6-feb2020.pdf

NSPOP=4

P(1)=0.068 ; PM

P(2)=0.376 ; IM

P(3)=0.525 ; EM

P(4)=0.031 ; UM

$ERROR

IPRED=F

Y=IPRED+IPRED*ERR(1)

AUC=A(4)

CAVG=AUC/TIME

TIMEABOVE=A(5)

CMAX1=COM(1)

TMAX1=COM(2)

$THETA

0.0417 ; 1 EM F1

18.8 ; 2 PM TO EM F1 RATIO

3.3 ; 3 IM TO EM F1 RATIO

0.434 ; 4 UM TO EM F1 RATIO

1.16 ; 5 CHRONIC DOSING PM ON F1

1.99 ; 6 CHRONIC DOSING NOT-PM ON F1

0.603 ; 7 D1

0.438 ; 8 KA

55.6 ; 9 CLBASE

40.3 ; 10 TM50 WEEKS

-9.09 ; 11 HILL

0.703 ; 12 PM ON CL

96.1 ; 13 VC

272 ; 14 VP

52.5 ; 15 Q

$OMEGA BLOCK(1)

0.195 ; 1 IOV F1

$OMEGA BLOCK(1) SAME

$OMEGA BLOCK(1) SAME

$OMEGA BLOCK(1) SAME

$OMEGA BLOCK(1) SAME

$OMEGA BLOCK(1) SAME

$OMEGA BLOCK(1) SAME

$OMEGA BLOCK(1) SAME

$OMEGA BLOCK(1) SAME

$OMEGA BLOCK(1) SAME

$OMEGA BLOCK(1) SAME

$OMEGA BLOCK(1) SAME

$OMEGA BLOCK(1) SAME

$OMEGA BLOCK(1) SAME

$OMEGA BLOCK(1) SAME

$OMEGA BLOCK(1) SAME

$OMEGA BLOCK(1) SAME

$OMEGA BLOCK(1) SAME

$OMEGA BLOCK(1) SAME

$OMEGA BLOCK(1) SAME

$OMEGA 0.717 ; 21 IIV F1

0.458 ; 22 IIV D1

0.0538 ; 23 IIV KA

0.0745 ; 24 IIV CL

0.3552 ; 25 IIV VC

$SIGMA 0.0001 ; DUMMY PROP ERR PK

62.4 ; ADD ERR QTC (MS)

$SIMULATION ONLYSIM SUBPROBLEMS=1 (3458) (322538)
